# The profound impact of COVID-19 on the control and care of diabetic patients: a comprehensive retrospective cohort study

**DOI:** 10.1186/s12875-024-02672-2

**Published:** 2024-12-21

**Authors:** Fakhria Al Rashdi, Salwa Al Harrasi, Mohammed Al Ismaili, AL-Ghaliya AL Yaaqubi, Zeenah Atwan, Celine Tabche

**Affiliations:** 1 Ministry of Health Sultan Oman, Muscat, Sultanate Oman; 2 School of Public Health / WHO Collaborating Centre/Department of Primary Care and Public Health/Imperial College London, Baghdad, Iraq; 3 School of Public Health / WHO Collaborating Centre/Department of Primary Care and Public Health/Imperial College London, London, UK

**Keywords:** COVID-19, Diabetes, Primary care, Oman

## Abstract

**Background:**

The COVID-19 pandemic has led to a significant shift in healthcare services, focusing on pandemic response and emergency preparedness. The Oman Ministry of Health implemented various measures to combat and control COVID-19. However, this shift disrupted routine outpatient appointments, particularly for chronic diseases such as diabetes mellitus (DM) and hypertension (HTN). This study aims to assess the pandemic’s effect on diabetes control, by examining glycated haemoglobin (HbA1c), blood pressure (BP), lipid values (particularly low-density lipoprotein (LDL), body weight/ body mass index (BMI), and comparing these measures to pre-pandemic levels.

**Methods:**

A retrospective cohort study of 223 people with diabetes (PwD), aged 20–95 years who underwent a blood workup in 2019 and 2020 and were registered in Al-Khuwair Health Centre from March to December 2020. Data was extracted from the Al Shifa 3plus System and National Diabetic Register (NDR), and analyzed using SPSS.

**Results:**

Out of 260 PwD identified, 223 met the inclusion criteria, while 37 were excluded due to recent diagnoses or missing follow-up in 2019. Significant changes were observed in HbA1C, systolic blood pressure (SBP), and BMI from 2019 to 2020. Mean HbA1c increased from 6.9% in 2019 to 7.2% in 2020. Mean SBP rose from 131.22 mmHg in 2019 to 134.84 mmHg in 2020, while mean BMI increased from 30.49 to 30.80. No significant changes were found in LDL levels or diastolic BP.

**Conclusion:**

The COVID-19 pandemic disrupted healthcare systems globally, and the consequences on health and mortality were not only due to the direct impact of the virus, but also to the modifications in priorities. These interruptions in inconsistent care, had consequences for non-communicable diseases (NCDs) like diabetes. Future strategic plans should be prepared and implemented to manage NCD cases in case of pandemics.

## Introduction

COVID-19 has severely affected the healthcare systems in low-, middle- and high-income countries and was declared a pandemic by the WHO. COVID-19 disrupted healthcare services, affecting cancer and TB screening, HIV detection, maternal health, children's vaccinations, and increasing mortality from non-communicable diseases (NCDs). In many countries, outpatient visits declined by 40% [1–4]. The pandemic had an impact on almost all health aspects, its impact was more significant on NCDs in almost all countries. Common NCDs, including diabetes, hypertension, cancer, asthma, and heart and kidney diseases, contribute to 7 of the top 10 major causes of premature death globally [5]. Approximately six months post-COVID-19 pandemic, the World Health Organization (WHO) assessed the impact of the pandemic on healthcare services for NCDs. The study revealed that of the 155 countries surveyed, “53% had partially or entirely disrupted healthcare services for treating hypertension, and 49% for treating diabetes and diabetes-related complications” [6] and an estimated 14% to 44% of COVID-19 patients had diabetes [5]. PwD who had SARS-CoV2 infection showed lower survival rates, poorer outcomes, prolonged hospitalization and higher mortality rates [7–9]. Furthermore, a comparison between patients with COVID-19 who are diabetic or not, showed a reduced chance of survival or recovery in PwD [5].

Worse clinical outcomes associated with SARS-CoV-2 infection in PwD, hypertension, liver conditions, chronic kidney and respiratory diseases could be attributed to upregulated expression of angiotensin-converting enzyme 2 ACE2 and cytokine storms. Ccomorbidities also increase the sensitivity to COVID-19, with diabetes increasing severity by up to 3%by diabetes [10], or the virus can be the cause to develop more severe outcomes [11]. A global survey found that 80% of chronic patients experienced worsening mental partly due to delayed or cancelled care appointments [12, 13].

A Brazilian study reported that significant number of PwD either avoided physical activities or postponed their apportionments, as a result, they experience a significant blood glucose disturbance [12]. However, patients depend either on home visits or on telemedicine to manage their blood glucose [5]. Similarly, in India some type 1 DM patients missed their insulin doses, blood glucose monitoring or compliance to the diet during lockdown [14]. Although telemedicine was employed to control blood glucose, 22% of PwD had an increase in their blood glucose levels [15]. Interstingly, some T1D showed improved glycemic control attributed to the more time for self-management [16].

Reduced acute physical activity negatively impacted insulin sensitivity lipid profiles, inflammation, and reduced muscle protein synthesis [17, 18] COVID-19 alters the lipid profile with lower TC, TG, LDL and HDL-C. These lower profiles are associated with the severity and mortality of the cases [19]. Other studies showed that total cholesterol and LDL were significantly higher post-lockdown compared to pre-lockdown [20]. Ratio of Urich acid to HDL-Cholestrol was revealed to be predictive to metabolic syndrome and possible type 2 diabetes [21]. Cumulative evidence from different countries showed a high prevalence of hypertension among patients with COVID-19 [22].

Oman was one of the countries affected by the pandemic, and accordingly, an impact on health services was noted. Most families have elderly members with NCDs. Some isolated them and reduced their visits to minimize SARS-CoV-2 exposure.

However, to what extent the disruption of DM care worsened the clinical outcomes was not very clear or evaluated. Therefore, it is important to evaluate the pandemic’s effects of COVID-19 on diabetic control concern (HbA1c), (BP), lipids particularly (LDL), and weight (BMI) and compare them with PwD pre-pandemic, to provide evidence-based recommendations for DM care during pandemics and prepare for sustainability of care in the future.

## Methods

### Study design and setting

This is a pre-and post-retrospective cohort study of adults with DM, who received follow-up in a primary care diabetes clinic.

### Population

All PwD registered in the DM Clinic et al.-Khuwair Health Centre were included. Omani patients aged over 18 years, diagnosed with type 1 or type 2 DM, with active follow-up and annual blood workups between January 2019 and December 2020. Active follow-up was defined as physical clinic visits or phone consultations with a blood workup.

Newly diagnosed type 1 & 2 DM patients or existing PwD without blood workups or follow-ups in 2019 were excluded.

The main outcomes of interest were the effect of COVID-19 related service modification and lockdowns on glycaemic control. Data were collected from the National Diabetic Register (NDR) and Al-Shifa System, managed by doctors working in ALKhuwair Health Centre, and analysed using SPSS with the help of a statistician.

### Outcomes of interest

This primary outcome was glycaemic control among patients, followed in the DM clinic, measured by determining patients’ clinical information extracted from the Al-Shifa System. This data included patients’ age, sex, DM risk factors, BMI, BP, HbA1c, and LDL. The latest follow-up data during the pandemic were used. All clinical parameters were measured using standardised policies and procedures across health centers to ensure accuracy and homogeneity.

#### Measurement of variables

- Blood HbA1c and LDL are measured in % and g/dL, respectively. General practitioners collected blood samples using standardised techniques and sent them to the laboratory for plasma analysis. The normal targets for PwD are < 7% for HbA1c and < 2.6 g/dL or < 1.8 for LDL in patients without and with cardiovascular comorbidities, respectively.
- BMI, measured in kg/m^2^. The measurement was performed by trained nurses using calibrated scales. The patient is considered underweight if their BMI is < 18.5 kg/m^2^, normal weight if their BMI is 18.5–24.9 kg/m^2^, overweight if their BMI is 25–29.9 kg/m^2^, and obese if their BMI is > 30 kg/m^2^.
- Blood pressure (BP) is measured in mmHg, by triage nurses using calibrated electronic measurement devices. The target BP in PwD is < 140/80 for those without complications and < 130/75 for those with cardiac and kidney disease.
- Data were collected through an electronic data collection sheet created using Google Forms.
- Age (continuous variable) and Sex (binary variable) were obtained from the NDR and adjusted for in the analyses
- Comorbidities (categorical variables) were extracted from the NDR.

### Statistical analysis

Data were analysed using SPSS. Frequency analyses using the mean, median, and percentage will describe primary outcomes. Clinical outcomes were compared using Chi-square tests.

### Data collection and management

After ethical approval from the regional research committee in Muscat.The study was performed in accordance with the Declaration of Helsinki. Researchers involved in the research retrieved the data using an electronic sheet, which was guaranteed confidentiality through a password set.

## Results

Out of 260 registered PwD who attended the DM clinic in 2019 and had a follow-up in 2020, 223 were included in the study, and 37 were excluded (new PwD and existing patients without follow-up in 2019). The population comprised 52% were male, and 48% were female with a mean age of 48.9%. Age distribution demonstrated in percentage in (Tables 1 and 2). Approximately96% of patients had type 2 DM with a mean duration of 10 years. All patients received DM service during the pandemic; 53.4% attended physical consultations, and 46.6% used phone consultations (Table 3).

**Table 1:** Study participant characteristics

	N (total *N* = 223)	Percent
Sex		
Male	116	52.0
Female	107	48.0
Age		
20–40 years	12	5.4
41–60 years	102	45.7
> 60 years	109	48.9

**Table 2:** Age percentages included in the study

	Frequency	Percent	Valid percent	Cumulative percent
Valid				
20–40 years	12	5.4	5.4	5.4
41–60 years	102	45.7	45.7	51.1
> 60 years	109	48.9	48.9	100
Total	223	100	100

**Table 3:** Consultations during the pandemic

	Frequency	Percent
Valid		
Physical	119	53.4
Phone	104	46.6
Total	223	100

Significant changes in HbA1c, systolic BP, and BMI were observed between 2019 and 2020.

The mean HbA1c in 2019 (6.9%) was significantly lower than in 2020 (7.2%), with a mean difference of − 0.30 (95% confidence interval [CI]: − 0.47– − 0.12; P = 0.0010) (Table 4).

**Table 4:** HbA1c values from 2019 to 2020

	HbA1c in 2019	HbA1c in 2020
*N*		
Valid	219	184
Missing	4	39
Mean	6.96	7.28
Median	6.70	6.80
Standard deviation	1.28	1.71
Minimum	4.30	4.88
Maximum	12.49	16.10

The mean SBP in 2019 (131.22 mmHg) was significantly lower than in 2020 (134.84 mmHg), with a mean difference of − 3.62 (95% CI: − 5.21– − 2.02; *P* = 0.0001).

The mean BMI in 2019 (30.49) was significantly lower than in 2020 (30.80), with a mean difference of − 0.31 (95% CI: − 0.54– − 0.08; *P* = 0.0090).

Other metabolic parameters did not change significantly from 2019 to 2020.

The mean LDL in 2019 (2.64) and 2020 (2.57) had a mean difference of 0.07 (95% CI: − 0.05–0.21; *P* = 0.2400). The mean diastolic BP in 2019 (78.10) and 2020 (78.21) had a mean difference of − 0.11 (95% CI: − 1.27–1.06; *P* = 0.856).

### Sex and HbA1c

A one-way analysis of covariance (ANOVA) was performed to assesswhether there was significant difference in HbA1c-2020 between males and females, adjusting for covariates HbA1c-2019 and DM duration. The results showed that males and females did not differ significantly in HbA1c-2020 after accounting for these covariates (*F*_(1, 177)_ = 3.187, *P* = 0.0760), Table 5.

**Table 5:** ANOVA of HbA1c-2020 by sex with HbA1c-2019 and DM duration as covariates

Source	Type III sum of squares	df	Mean square	*F*	*P*	Partial η^2^
HbA1c-2019 (covariate)	284.336	1	284.336	208.444	0.000	0.541
DM duration (covariate)	0.760	1	0.760	0.557	0.456	0.003
**Sex**	4.347	1	4.347	3.187	**0.076**	0.018
Error	241.444	177	1.364			

*Key*: *df* degrees of freedom

Unadjusted *R*^2^ = 0.548. Adjusted *R*^2^ = 0.540

### Age and HbA1c

A one-way ANOVA was performed to assess the significance of differences between the three age groups in HbA1c-2020 when adjusting for covariates HbA1c-2019 and DM duration. The results showed that the three age groups didn’t differ significantly in HbA1c-2020 after adjusting for covariates (*F*_(2, 176)_ = 1.881, *P* = 0.1555.

Table 6.

**Table 6:** ANCOVA of HbA1c-2020 by age with HbA1c-2019 and DM duration as covariates

Source	Type III sum of squares	df	Mean square	*F*	*P*	Partial *η*^2^
HbA1c-2019 (covariate)	252.690	1	252.690	184.809	0.000	0.512
Duration of Diabetes (covariate)	0.802	1	0.802	0.587	0.445	0.003
**Age**	5.145	2	2.572	1.881	**0.155**	0.021
Error	240.646	176	1.367			

*Key*: *df* degrees of freedom

Unadjusted *R*^2^ = 0.549. Adjusted *R*^2^ = 0.539

## Discussion

This paper aimed to provide evidence on the impact of COVID-19 on diabetic control, including HBA1C, LDL, BP, and BMI, in PwD attending Alkhuwair Health Centre, comparing 2019 (pre-COVID) and 2020 (post-COVID).

The results showed statistically significant HbA1c, systolic BP, and BMI changes between 2019 and 2020. The mean HbA1c in 2019 (6.9%) was significantly lower than in 2020 (7.2%). After resuming the clinic, it was noticed that some patients had higher glycated haemoglobin (HbA1c) levels than last year.

SARS-CoV2 virus could interfere with the glucose metabolism pathways by triggering the reprogramming of the glucose metabolism through AMP activating protein kinase with no effect on the pancreas [23].

Diabetes causes metabolic and vascular changes,inhibiting the innate immune response and releasing inflammatory cytokines [24]. This hyperinflammation can result in multiple organ failure and endothelial dysfunction [25, 26] So COVID-19 can worsen existing diabetes or trigger diabetes in non-diabetic individuals [24].

The HBA1c results in this study are consistent with a study that found that HbA1c values significantly increased from 7.45% to 7.53% during the pandemic [27]. However, it was found that despite the lockdown, there were improvements in glycaemic control in patients with type 1 DM due to self-management [16]. Both sex and age groups did not differ significantly in our study regarding HbA1c in 2020. These results were inconsistent with the conclusion of Tanji et al., who noticed that the deterioration in HbA1c values was more apparent in women, patients aged ≥ 65 years, patients with BMI > 25, and patients not using insulin [27].

The mean BMI in 2019 (30.49) was significantly lower than in 2020 (30.80). This finding can be explained by the lockdown, reducing physical activity, and changing lifestyle habits which lead to raising awareness about the importance of nutritional status for a healthy lifestyle [28, 29]. Considering the extreme weight categories associated with severe COVID-19 complication risk, we noticed that 13.11% of the total sample fell into these vulnerable categories. Being overweight or obese is an independent risk factor in severe COVID-19 patients because enhanced adiposity diminishes pulmonary function [29]. Healthy lifestyles and choices should be promoted in primary care centres with the help of multidisciplinary teams. Obesity and overweight rates are on the rise, especially in the eastern Mediterranean region, which will cause a further burden on our health systems [30].

Another factor that could be implicated here is stress, anxiety, and isolation, especially for older people, which was reported during the COVID-19 pandemic [29, 31]. Stress is an important factor implicated in the dysfunctionality of the sympathetic nervous system and the hypothalamus that leads to obesity. The other consequence of stress is the tendency to develop eating disorders and lower physical activity. All these factors might explain the increase in BMI noticed in the study cohort [32]. Positive relationships were found between dealing with stress related to COVID-19 in patients with NCD and active coping strategies, for example, self-distraction, denial, substance use, behavioral disengagement, venting, planning, religion, and self-blame [33].

In addition to HBA1c and weight, clinic patients showed high systolic BP and LDL. The results are in line with Akpek 2020, which suggests that infection with SARS-CoV-2 increases systolic and diastolic BP and could lead to hypertension [34]. In our study, only systolic BP was significantly higher, but diastolic BP was not. However, the results contradict Feitosa et al., who showed no considerable adverse impact of COVID-19 on office and home BP [35].

Many studies considered an association between antihypertensive medication classes and patient outcomes, but almost all are retrospective investigations or meta-analyses. Therefore, well-conducted research with a considerable number of hypertensive patients is necessary to resolve current controversies about the relationship between hypertension and COVID-19 [34, 36]. Aras Júnior et al. showed that hypertension is a significant risk factor for mortality, and it contributes to greater COVID-19 severity, intensive care unit admission, and mortality with age, other cardiovascular risk factors [37].

Telemedicine consultation existed at a comparable percentage to physical consultation, which is attributed to the fact that in Oman, the health system, like other countries, adopted many changes in NCD routine management [38–40]. The Directorate of General Health Services in Muscat implemented a telemedicine clinic twice weekly for patient follow-up and consultation in the primary care setting. So, the results align with Chudasama et al., in 2020, which showed that 45% of the participants’ healthcare providers performed telephone [13]. Furthermore, a WHO survey of 155 countries found that 58% now use telemedicine to replace in-person consultation [6].

### Limitations

Residual confounding can be a challenge in observational studies. To address this, we should include as many confounders as possible in the regression analysis and seek the opinions of clinical experts. Additionally, sensitivity analysis was employed to evaluate any hidden residual confounding, such as mental instability. Negative outcome control could be applied to explore any hidden confounding due to measurement errors for lifestyle factors. Missing data is a major potential limitation. We tried to locate and reference relevant sources of patient information to retrieve any missing data from electronic health records. Furthermore, we performed a sensitivity analysis to manage the missing data. Selection bias was another concern. Further, this is a single-centre study, so it does not reflect the whole of Oman. It is recommended that future similar research have a bigger sample size from many health centres. Lastly, some of the Omani population receive medical care in private institutions that are not registered in the national registries.

## Conclusion

The COVID-19 pandemic is ongoing with new emerging strains, and many studies have been published exploring COVID-19’s impact on different diseases. In this study, we can conclude that there was a significant adverse effect on glycaemic control in DM patients. This finding suggests that we must provide extra care to patients with non-communicable diseases under normal conditions to prepare them to face such future challenges. This can be achieved by promoting healthy lifestyles and ensuring that our healthcare systems advocate for healthy environments where people can practice a healthy lifestyle and access nutritious foods and activity centres. Additionally, having primary care centres as their support can further facilitate this goal.

### Limitations

This retrospective study was conducted in (Al-Kuwair Health Center) which may not represent the whole Omani population. Mental health, changes in lifestyles and how patients adhered to their medication program during the pandemic were not included. For future directions, the data could be strengthened through combining data from different health centers to make it possible to generalize the conclusion.

## Data Availability

The datasets used and/or analyzed during the current study are available from the corresponding author on reasonable request.

## Abbreviations

DM: Diabetes mellitus
HTN: Hypertension
PwD: People with diabetes
HbA1c: Glycated haemoglobin
BP: Blood pressure
LDL: Low-density lipoprotein
BMI: Body mass index
SPSS: Statistical Package for the Social Sciences
NDR: National Diabetic Register
NCDs: Non-communicable diseases
WHO: World Health Organization
ANOVA: A one-way analysis of covariance
